# Human induced pluripotent stem cell-derived microglia with 1q21.1 deletion and duplication exhibit aberrant inflammatory response

**DOI:** 10.1016/j.gendis.2025.101923

**Published:** 2025-11-06

**Authors:** Tanya Singh, Kubra Trabzonlu, Aleksandra Spasova, Phil Stephens, David E.J. Linden, Marianne B.M. van den Bree, Michael J. Owen, Jeremy Hall, Ujjwal Neogi, Adrian J. Harwood, Yasir Ahmed Syed

**Affiliations:** a Neuroscience and Mental Health Innovation Institute, Hadyn Ellis Building, Cardiff University, Cathays, Cardiff CF24 4HQ, UK; b School of Bioscience, Cardiff University, The Sir Martin Evans Building, Museum Ave, Cardiff CF10 3AX, UK; c School of Dentistry, Cardiff University, Cardiff CF14 4XY, UK; d Mental Health and Neuroscience Institute (MHeNs), Faculty of Health, Medicine and Life Sciences, Maastricht University, Maastricht 6229 ER, the Netherlands; e Division of Psychological Medicine and Clinical Neurosciences (DPMCN), School of Medicine, Cardiff University, Cardiff CF14 4YS, UK; f Centre for Neuropsychiatric Genetics and Genomics, Hadyn Ellis Building, School of Medicine, Cardiff University, Cardiff CF24 4HQ, UK; g The Systems Virology Lab, Division of Clinical Microbiology, Department of Laboratory Medicine, Karolinska Institutet, Huddinge 141 52, Sweden

Neuroinflammation plays a significant role in neurodevelopmental and neuropsychiatric disorders (NPDs), such as autism spectrum disorder, schizophrenia, and major depressive disorder. Microglia, the resident immune cells of the central nervous system, are essential for brain development, engaging in neurogenesis, synaptic pruning, and immune surveillance. Elevated cytokine levels and gene expression, leading to increased microglial activity, have been previously detected in autism spectrum disorder patients, suggesting a link with certain copy number variants (CNVs).^1^ The role of CNVs in the progression of NPDs linked to microglial dysfunction remains largely unknown. Therefore, we generated an *in vitro* microglial model system from patient-derived induced pluripotent stem cells (iPSC) carrying a deletion or duplication at the 1q21.1 chromosomal region, previously associated with developmental delay, autism spectrum disorder, and schizophrenia. Our study demonstrates a morphological dissimilarity, dysregulation in microglial cytokine production, and responsiveness in the presence of 1q21.1 CNV. Furthermore, our findings underscore the up-regulation of key genes regulating the inflammation and immune response, such as *ICAM1*, *TRAF6*, *STAT3*, and *IFNRG1* in 1q21.1 CNV, suggesting a link between immune dysfunction and genetic abnormality at the cellular level.

To investigate the impact of microglia in the presence of 1q21.1 CNVs, we differentiated iPSCs from patients with deletion or duplication into microglia-like cells, following a previously established protocol^2^ (Fig. 1A; supplementary material). The microglia with 1q21.1 CNVs exhibited morphological abnormalities, with elongated structures and reduced branching compared with control (Fig. 1B). However, microglial-specific calcium-binding protein *IBA1* did not reveal a difference in the expression levels of *IBA1*-positive (*IBA*^*+*^) cells among iPSC-derived microglia (Fig. 1B). To further validate the observed morphological changes, the number of *IBA1*^+^ cells was assessed in the hippocampal region of a mouse model with 1q21.1 deletion.^3^ The *in vivo* immuno-labelling of 1q21.1 deletion mouse showed a significantly lower number of *IBA1*^+^ microglia (Fig. 1C), suggesting that the difference between *in vitro* and *in vivo IBA1* expression might be due to the presence of 1q21.1 CNV impacting microglial maturation during early brain development. Next, we investigated changes in synaptic protein expression within the 1q21.1 mouse model and identified a reduction in the postsynaptic protein PSD95 (Fig. S4). These findings align with Chapman et al (2022),^4^ who reported similar disruptions in synaptic morphology and protein expression in a 1q21.1 deletion model derived from patient cortical neurons. Collectively, these results suggest that microglial dysfunction may play a critical role in abnormal synaptic development *in vivo*.

**Figure 1:**
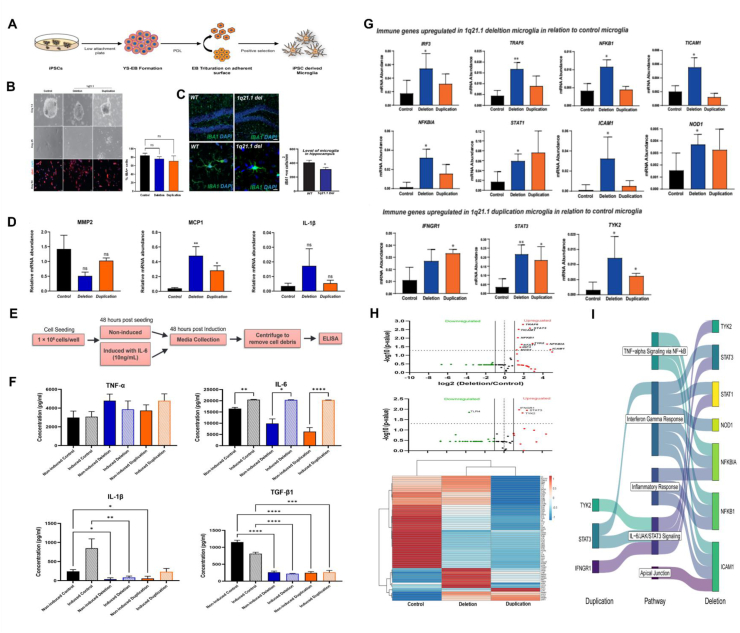
Association between microglial dysregulation and 1q21.1 copy number variants (CNVs). **(A)** Microglia differentiation from induced pluripotent stem cells (iPSCs). The iPSCs were differentiated to embryoid bodies (EBs) for 14 days and then transferred to poly-d-lysine (PDL)-coated plates. After positive selection on day 14, the microglia-like cells began to form**. (B)** Characterisation of iPSC-derived microglia. In the top panel, day 14 illustrates EBs' morphology in suspension culture, while day 45 shows mature microglia. The bottom shows the representative fluorescent images of day 50 microglia. Microglia expressing *IBA1* (red) were counterstained with *DAPI* (blue). Scale bar: 50 μm. The bar graph illustrates the percentage of IBA^+^ cells in control and 1q21.1 CNV microglia. The data were presented as mean ± standard error of the mean. Control: *n* = 2; 1q21.1 deletion: *n* = 2; 1q21.1 duplication: *n* = 3. **(C)** Microglial cells from the hippocampal region of a mouse model carrying a 1q21.1 deletion. The fluorescent images visualise IBA1^+^ cells (green) in the hippocampus of a 4-week-old mouse brain, counterstained with DAPI (blue). Scale bar: 50 μm. The graph illustrates a comparison of the IBA1+ve microglial population in the hippocampal region of control and 1q21.1 deletion mice. The data were represented as mean ± standard error of the mean. Control: *n* = 6; 1q21.1 deletion: *n* = 6. **(D)** Gene expression of matrix metalloproteinase (MMP)-related genes. The bar graph illustrates the gene expression of microglial genes (*MMP2*, *MCP1*, and *IL-1β*). The data were presented as mean ± standard error of the mean. Control: *n* = 2; 1q21.1 deletion: *n* = 3; 1q21.1 duplication: *n* = 3. **(E)** Schematic workflow of ELISA, which was employed to determine the cytokine profile in microglial cells. A total of 0.1 × 10^6^ cells/well were seeded in a 24-well plate and allowed to attach for the next 48 h. Subsequently, the cells were induced with 10 ng/mL IL-6, and conditioned media were collected 24 h post-induction. Simultaneously, the non-induced control was also collected at the same time. **(F)** Bar graph of the cytokine levels (*TNF-α*, *IL-6*, *IL-1β*, *TGF-β1*) in resting microglia and those induced with IL-6 (10 ng/mL) as measured by ELISA. The data were shown as mean ± standard error of the mean. Control: *n* = 2; 1q21.1 deletion: *n* = 3; 1q21.1 duplication: *n* = 3. **(G)** Bar graph of the gene expression of immune genes showing up-regulation in 1q21.1 deletion (*IRF3*, *TRAF6*, *NFKB1*, *TICAM1*, *NFKBIA*, *STAT1*, *ICAM1*, and *NOD1*) and 1q21.1 duplication (IFNGR1, STAT3, and TYK2) microglia compared with the control. The data were represented as mean ± standard error of the mean. Control: *n* = 2; 1q21.1 deletion: *n* = 2; 1q21.1 duplication: *n* = 2. The *p*-values were calculated using a student's *t*-test of the replicate ΔCT method values for each gene in the control group and 1q21.1 CNV groups. **(H)** Visual representation of the pathway analysis. All gene expression analyses were normalized with GAPDH using the ΔCT method. **(I)** Sankey plot of inflammation-associated biological processes in microglia with 1q21.1 deletion and duplication. Statistical analysis was performed using the Kruskal–Wallis test comparing the duplication/deletion to the control, followed by Dunn's multiple comparisons test, unless specified. Significance levels were denoted as ns (*p* > 0.05), ∗*p* < 0.05, ∗∗*p* < 0.002, ∗∗∗*p* < 0.0002, and ∗∗∗∗*p* < 0.0001.

To further explore how 1q21.1 CNV affects the expression of immune-related genes during microglial development, we performed gene expression analysis. Prior research suggests that matrix metalloproteinases (MMPs) are linked to heightened inflammatory responses in NPDs. MMPs are known to activate inflammatory signalling pathways, further influencing microglial behaviour. Our results revealed that the chemokine *MCP1*, which plays a crucial role in microglial activation and neuroinflammation, was significantly up-regulated in 1q21.1 CNVs, especially in deletion (Fig. 1D). Although statistically insignificant, we still observed down-regulation of *MMP2* and *IL-1β* in both 1q21.1 CNVs. These findings suggest that 1q21.1 CNVs not only impact microglia development but also influence the expression of immune-related genes.

Microglia activation is vital in tissue repair and restoring brain homeostasis. This process can be triggered by pathological conditions *in vivo* or by stimulating the microglia with proteins, cytokines, and chemical agents, both *in vivo* and *in vitro.* Therefore, we validated the functionality of the iPSC-derived microglia through ELISA (Fig. 1E). After 24 h of treatment with *IL-*6 cytokine (10 ng/mL), the degree of microglial activation was assessed trough analyses of the extracellular levels of pro-inflammatory cytokines, such as *IL-1β*, *TNF-α*, and *IL-6*, and anti-inflammatory *TGF-β1* in the microglial conditioned media from both the resting and activated experimental groups. There were no significant differences in the expression levels of *TNF-α* before and after the microglial induction, nor between the 1q21.1 CNV lines and the controls (Fig. 1F). In contrast, the expression of *IL-6* was lower in microglial cells with non-induced 1q21.1 deletion and duplication, when compared with treated 1q21.1 CNV microglia. Although the expression of *IL-6* was lower in microglial cells with 1q21.1 deletion and duplication before induction, this difference did not reach statistical significance compared with the non-induced controls (Fig. 1F). Notably, following induction, the level of *IL-6* reached the maximum limit of the ELISA kit for measurement, potentially due to the presence of *IL-6* used for induction. The activation of the induced microglial-like cells resulted in a significant overexpression of the cytokine *IL-1β* only in the induced control group (Fig. 1F), suggesting that when microglia are not active, the *IL-1β* expression is low. The pro-inflammatory cytokines *IL-1β*, *TNF-α*, and *IL-6* are linked to induce the NF-κB signalling pathway, a key regulator in neuronal differentiation, synaptic growth, and neuroinflammation.^5^ Therefore, these findings suggest that microglial cells with 1q21.1 deletion and duplication may have a compromised ability to activate and secrete the necessary levels of cytokines required for their physiological function and neuronal function.

To further examine the impact of the 1q21.1 CNVs in microglia on inflammatory pathways, we conducted pathway analysis utilising RT^2^ Profiler™ PCR array to assess key neuroinflammatory (Table S5). The results from GeneGlobe indicated that the 1q21.1 microglia exhibited significant changes in genes related to the inflammatory response, as well as innate and adaptive immunity. Specifically, *IRF3*, *TRAF6*, *NFKB1*, *TICAM1*, *NFKBIA*, *STAT1*, *ICAM1*, and *NOD1* genes were up-regulated in microglia with 1q21.1 deletion, whereas *IFNGR1*, *STAT3*, and *TYK2* genes were up-regulated in microglia with 1q21.1 duplication (Fig. 1G and H). The gene set enrichment analysis of the significantly up-regulated genes using the Molecular Signatures Database (MSigDB) identified activation of NF-κB, interferon, and IL-6/STAT3 pathways (Fig. 1I), which suggests heightened neuroinflammation, driving microglial activation and cytokine production. These results confirmed that microglia with 1q21.1 deletion or duplication were associated with dysregulated immune gene expression, reflecting an inflammatory milieu that could contribute to neuronal dysfunction.

Previous research demonstrated the significance of TRAF6's role in inflammatory response and microglial activation, demonstrating that TRAF6 down-regulation suppresses the *NF-κB* signalling pathway, impacting microglial polarisation. Therefore, our findings suggest that microglia carrying 1q21.1 CNV may suppress inflammatory signalling downstream of the *TRAF6/NF-κB* cascade (Fig. S5).

In a healthy brain, microglial *NF-κB* signalling is essential to maintain homeostasis and neuronal integrity. Previously, it has been demonstrated that the down-regulation of the pathway leads to neuronal apoptosis in the forebrain, whereas up-regulation has the opposite effect.^5^ Additionally, *NF-κB* and *STAT3* crosstalk regulates cytokine expression and immune responses, suggesting that 1q21.1 CNV influences this signalling axis, potentially linking it to NPDs.

A limitation of this study is the absence of a peripheral inflammation model, which plays a critical role in triggering microglial activation via cytokine release. The blood–brain barrier is necessary during peripheral inflammation, allowing immune molecules to reach the brain, influencing microglial responses. Investigating these interactions in a 1q21.1 CNV model is essential to understanding how peripheral immune signals contribute to NPDs. Additionally, interactions between microglia and other brain-resident cells should be further explored to elucidate their roles in microglial activation under inflammatory conditions.

Moreover, pathway analysis using QIAGEN RT^2^ Profiler Data Analysis Software provided a list of differentially expressed genes in 1q21.1 CNV microglia compared with controls. Further, we could not find a direct association of the 1q21.1 locus gene directly to the inflammation pathway. It is likely that the dosage combination of locus genes is likely to influence the inflammatory pathway (Fig. S3). While these findings offer an initial framework for understanding molecular disruptions, further research should incorporate broader analysis involving pharmacological validation, proteomic signature, DNA methylation, transcription factor activity, and sex differences.

In summary, our study highlights the intricate relationship between the 1q21.1 chromosomal locus and microglial function. Mutations at this locus lead to significant effects, including morphological changes, disrupted immune-related gene expression, impaired activation capacity, and dysregulated immune responses in microglia. These findings underscore the multifaceted impact of 1q21.1 deletion and duplication on microglial function. By uncovering these mechanisms, our research provides valuable insights into how high-risk genetic CNVs contribute to neuroinflammatory and neuropsychiatric disorders.

## CRediT authorship contribution statement

**Tanya Singh:** Writing – original draft, Visualization, Methodology, Investigation, Formal analysis. **Kubra Trabzonlu:** Methodology, Investigation. **Aleksandra Spasova:** Writing – review & editing, Writing – original draft. **Phil Stephens:** Resources. **David E.J. Linden:** Resources. **Marianne B.M. van den Bree:** Resources. **Michael J. Owen:** Resources. **Jeremy Hall:** Resources. **Ujjwal Neogi:** Formal analysis. **Adrian J. Harwood:** Resources. **Yasir Ahmed Syed:** Conceptualization, Writing – original draft, Supervision, Project administration, Methodology.

## Ethics declaration

The generation and use of iPSCs for the study was approved by Cardiff University and the Health and Safety Executive (GMO130/19.3). The methods of generation of iPSC lines followed the guidelines of the approving committee. Clinical and psychometric testing of participants were approved by the Local Research Ethics Committee of the National Health Service (study 14/WA/0035). Written consent was obtained from all participants.

## Funding

This work was supported by a CMU fellowship, Hodge Foundation and Medical Research Foundation grant to Y.A.S.; A.J.H. and J.H. are supported by the 10.13039/100012107TWF Changing Minds Program; J.H., A.J.H, D.E.J.L., and M.O. were supported by 10.13039/100004440Wellcome (DEFINE Strategic Award 100202/Z/12/Z); J.H. is also supported by Hodge Foundation (Centre Grant) and 10.13039/501100000265MRC grants (No. MR/L010305/1, MR/NO22572/1, MR/L011166/1) and 10.13039/501100001313CU grant (No. G0800509); M.B.M.B. is supported by grants from the 10.13039/501100000265MRC (No. MR/W014416/1, MR/W028395/1, MR/W020297/1, MR/T033045/1, MR/S037667/1), NIMH (No. U01MH119758), the Wellcome Trust (No. 226709/Z/22Z), and Welsh Government (HS 22 04). The work was also supported by the core facilities of the Neuroscience and 10.13039/100011705Mental Health Research Institute, 10.13039/501100000866Cardiff University, UK, and the National Centre for Mental 10.13039/100018696Health.

## Conflict of interests

The authors declared no conflict of interests.
