# Supplementary material for: Human induced pluripotent stem cell-derived microglia with 1q21.1 deletion and duplication exhibit aberrant inflammatory response
Source: Genes Dis. 2025 Nov 6;13(4):101923. doi: 10.1016/j.gendis.2025.101923 (PMC13011030; doi:10.1016/j.gendis.2025.101923)
Supplement: Multimedia component 1 [file mmc1.docx]

**Materials and Methods**

**Ethics statement**

The generation and use of iPSCs for the study was approved by Cardiff University and the Health and Safety Executive (GMO130/19.3). The methods of generation of iPSC lines followed the guidelines of the approving committee. Clinical and psychometric testing of participants were approved by the Local Research Ethics Committee of the National Health Service (study 14/WA/0035). Written consent was obtained from all participants. There were commercially produced 2 control lines (IBJ4, see Plumby et al. ^1^ and HPSI1013i-wuye_2 purchased from HipSci), and mutation lines derived from patient samples – 3 deletion lines and 3 duplication lines used for this study, unless specified (Fig. S1, S2, Table S1, see Champan, et al, 2022^2^ for all deletions and duplication 1 and 2). The lines used were characterized and have been published previously by Chapman, et al, 2022 ^2^. One additional duplication line (Duplication 4, see Fig. S1) has been used in this study which was derived from the Neurosciences Mental Health Research Institute iPSC core facility (Cardiff University).

**Microglial differentiation from iPSCs**

Microglia differentiation was adapted from protocols by Muffat, et al, 2016^3^. iPSCs were maintained on Geltrex™ coated plates in E8 Flex media (Gibco™, A2858501). iPSCs were passaged using Versene solution (Gibco™ 15040066) and gently triturated to form a suspension of uniform clumps. The microglia-like cells were generated from iPSCs using a differentiation protocol previously described by Muffat et al, 2016^3^ (Fig. S1). The clusters were cultured in Microglial differentiation media (MGdM) see Table S2 supplemented with 10ng/mL IL-34 and 10ng/mL of Colony-Stimulating Factor 1 (CSF1), in an ultra-low attachment plate. Embryoid bodies (EBs) were monitored for 14 days and then further transferred on poly-D-lysine coated plates. For the next 30 days (every 5th day), EBs were gently triturated ^3^ to shear off loose cells of interest and positively selected and seeded on Primaria™ plates. Unattached cells and small EBs were washed with fresh MGdM. The attached cells were monitored for morphological characteristics of microglia.

**Construction of 1q21.1 deletion mice model**

The detailed construction strategy for this model was published in Nielsen et al, 2017^4^.

**Immunocytochemistry (ICC)**

Cells were incubated with 4% paraformaldehyde (PFA) for 10 minutes at room temperature (RT). PFA was then removed, and cells were washed with PBS. For blocking and permeabilization, cells were further incubated in 3% donkey serum with 0.03% Triton-X-100 for an hour at RT. After three PBS washes, cells were incubated overnight at 4°C in blocking solution and primary antibodies IBA1 (1:500; Wako), CD40 (1:500; eBioscience), SYN (1:400, Abcam), PSD95 (1:800, Abcam). The next day the primary antibody solution was removed, and cells were washed 3 times with PBS. Cells were then incubated for 1 hour at RT in 3% goat serum and secondary antibodies conjugated to Alexa Fluor 594 (1:2000; Life Technologies). All the antibodies used in this study are represented Table S3. The secondary antibody solution was then removed, and the cells were washed three times with PBS followed by counterstaining with DAPI and mounting using Fluoromount Mounting Medium (Sigma). Imaging was undertaken on a Leica DMI600 Inverted Microscope. For cell counting purposes, a minimum of two biological repeats and 5 random fields were selected for imaging.

**Quantitative Real-Time PCR (qRT PCR)**

Cells were dissociated using Accutase and the cell suspension was centrifuged at 200 RCF (10 000 x g) for 5 minutes at RT. The supernatant was discarded, and the cell pellet was used for RNA isolation. RNA was isolated using a GeneElute mammalian total RNA miniprep kit (Sigma) with on-column DNase treatment and was eluted in 20μl of nuclease-free water. The concentration of RNA measured on a nano-drop machine was used to calculate 500ng of RNA for cDNA synthesis. The cDNA was synthesized using a high-capacity cDNA reverse transcription kit (Applied Biosystems) using the manufacturer's protocol. 100ng of cDNA was used for 20 μl of qRT-PCR reaction. The primers sequences were either prepared on Primer Blast or previously reported (Table S4). The qRT-PCR reactions were run on a StepOnePlus real-time PCR system (Applied Biosystems) with SYBR green, qPCRBIO SyGreen Blue Mix Hi-ROX (PCR Biosystems). Each run was performed with three technical replicates for every biological sample. The threshold cycle (CT) value during the log phase of amplification was normalized to the expression of the housekeeping gene – GAPDH. The cutoff for CT value was 30. Results are calculated and presented either as mRNA abundance (2−ΔCT) or as fold change in gene expression (2−ΔΔCT) method.

**Enzyme-linked Immunosorbent Assay (ELISA)**

500 µl MGdM medium (serum-free) was added to microglia cell culture. The medium was collected from wells 24 hours post-feeding and centrifuged at 200 RCF (10 000 x g) for 5 minutes to get rid of remaining cell debris. Finally, the supernatant was collected for either same-day use or stored at -20⁰C until further use. Cytokine secretion into the microglia culture medium was measured using Invitrogen™ Human Uncoated ELISA Kit, according to the manufacturer’s protocol. The absorbance of the experimental and control wells of the microplate was measured in the fluorescence plate reader at 450nm with appropriate sensitivity settings.

**Pathway Analysis**

Pathway analysis was undertaken utalising RT² Profiler PCR Arrays (Qiagen). Each catalogued RT² Profiler PCR Array contains a list of pathway-focused genes as well as five housekeeping genes (Table S5). In addition, each array contains a panel of proprietary controls to monitor genomic DNA contamination (GDC) as well as the first strand synthesis (RTC) and real-time PCR efficiency (PPC). The qPCR Assays used in PCR Arrays are laboratory-verified and optimized to work under standard conditions enabling a large number of genes to be assayed simultaneously. Their specificity is guaranteed when RT² SYBR Green qPCR Master Mixes are used as part of the complete PCR Array System protocol. In this study, 96 genes were profiled on 12 samples with the PAHS-052Z (RT² Profiler™ PCR Array Human Innate & Adaptive Immune Responses). RNA was isolated using an RNA extraction kit according to the manufacturer’s instructions and quantified using a nanodrop machine. The RNA was then reverse transcribed using a cDNA conversion kit (RT² First Strand Kit, 330401). The cDNA was used on the real-time RT² Profiler PCR Array (QIAGEN, Cat. no. PAHS-052Z) in combination with RT² SYBR® Green qPCR Mastermix (Cat. no. 330529). CT values were exported to an Excel file to create a table, which was later uploaded to the data analysis web portal at http://www.qiagen.com/geneglobe. Samples were assigned to controls and test groups. T values were normalized based on a Manual Selection of reference genes. The data analysis web portal calculates fold change/regulation using the Δ-Δ CT method, in which Δ CT is calculated between the gene of interest (GOI) and an average of reference genes (HKG), followed by Δ-Δ CT calculations (Δ CT (Test Group)-Δ CT (Control Group)). Fold Change is then calculated using the 2^(-Δ-Δ CT)^ formula. The data analysis web portal also plots scatter plot, volcano plot, cluster gram, and heat map. This data analysis report was exported from the QIAGEN web portal at GeneGlobe.

**Statistical analysis**

The reproducibility experiments were performed with an independent set of differentiation for each cell line and 3 technical replicates for each set of differentiations. Statistical analysis was performed using GraphPad prism version 9.00 (GraphPad Software; La Jolla, CA, USA). The statistical tests used for data analysis were specific to experiments and are detailed in the figure legends. N indicates number of control, deletion and duplication cell lines and n denotes the sets of differentiations used in each analysis. For pathway analysis, 2 control lines, 2 deletion lines and 2 duplication lines were used with 2 sets of differentiation each. The RT² Profiler PCR Arrays analysis was done using QIAGEN web portal at GeneGlobe (http://www.qiagen.com/geneglobe) as recommended by manufacturer.

**Supplemental References**

1. Plumbly W, Brandon N, Deeb TZ, Hall J, Harwood AJ. L-type voltage-gated calcium channel regulation of in vitro human cortical neuronal networks. *Scientific Reports*. 2019;9(1):13810.

2. Chapman G, Alsaqati M, Lunn S, et al. Using induced pluripotent stem cells to investigate human neuronal phenotypes in 1q21. 1 deletion and duplication syndrome. *Molecular Psychiatry*. 2022;27(2):819-830.

3. Muffat J, Li Y, Yuan B, et al. Efficient derivation of microglia-like cells from human pluripotent stem cells. *Nature medicine*. 2016;22(11):1358-1367.

4. Nielsen J, Fejgin K, Sotty F, et al. A mouse model of the schizophrenia-associated 1q21. 1 microdeletion syndrome exhibits altered mesolimbic dopamine transmission. *Translational psychiatry*. 2017;7(11):1261.


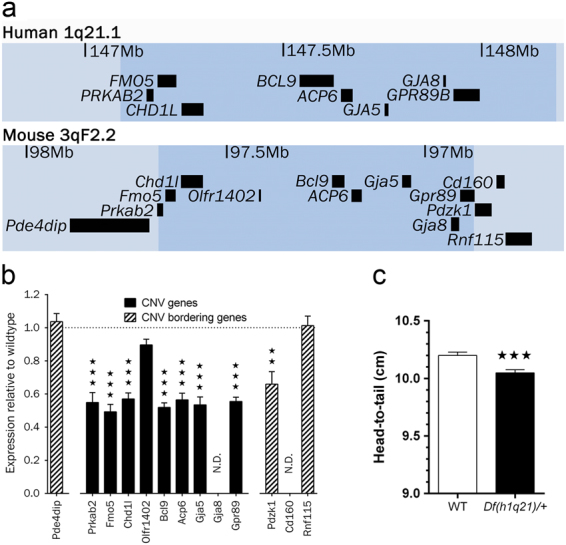
**Material and Methods Supplementary Figures**

**
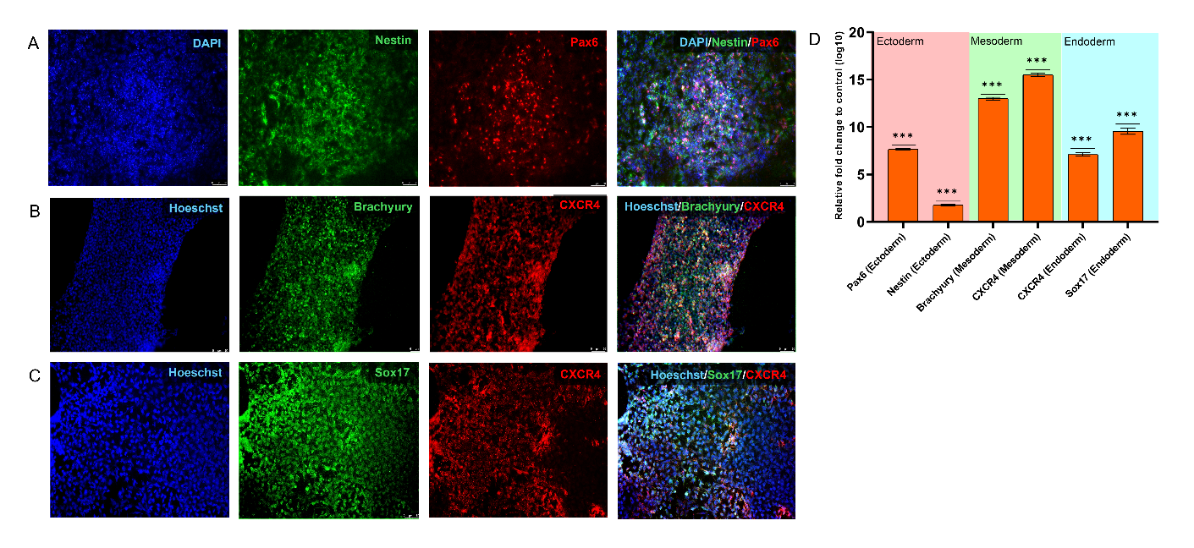
Supplementary Figure 1. Visualization of the construction similarities between *Df(h1q21)/* *+* mice and human deletion carriers. a.** Overview of the delated region in mouse model and human carrying 1q21.1 deletion. **b.** Gene expression changes from frontal cerebral cortex in 14-week-old 1q21.1 deletion mouse compared with wild-type. **c.** Head-tot-tail base length decrease (p<000.1) in 1q21.1 deletion mice. The figure is taken from Nielsen et al, 2017^4^.

**Supplementary Figure 2. Duplication line 4 with 1q21.1 mutation coordinates - chr1:146326373-147385641**. **A**. Representative images for DAPI, Nestin, Pax6 positive markers in the Ectoderm. **B**. Representative images for Hoeschst, Brachyury, CXCR4 in the Mesoderm. **C**. Representative images for Hoeschst, Sox17, CXCR4 positives markers in the Endoderm. **D.** Trilineage Fold Change graph. presenting relative fold change of the duplication cell line compared to the control. The data shown present the relative fold change of the Duplication 4 cell line compared to the control, ns p > 0.05, * p<0.05, ** p<0.005, *** p<0.0005 and **** p<0.0001 using Kruskal–Wallis test, Dunn’s multiple comparisons test.


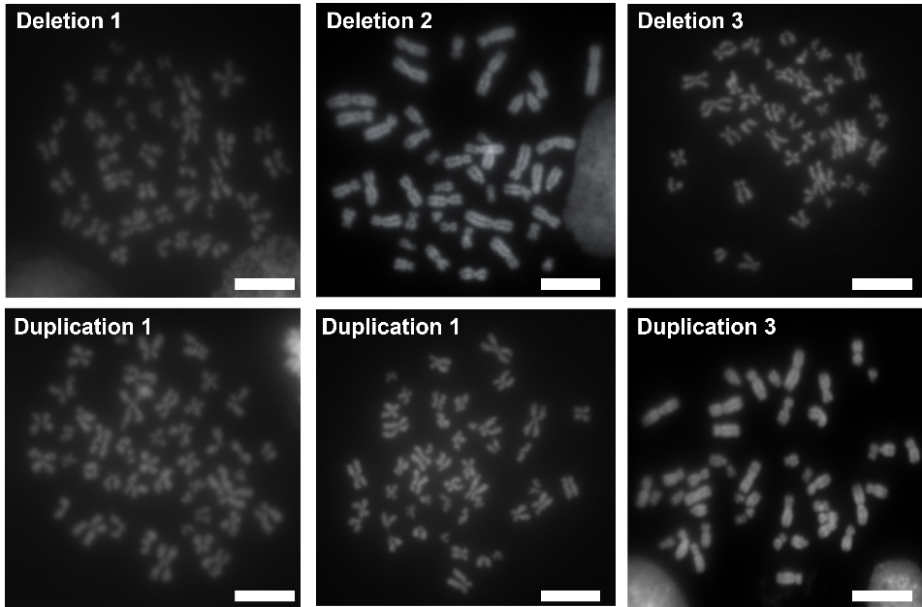


**Supplementary** **Figure 3. Representative brightfield images of metaphase spreads from 1q21.1 deletion and 1q21.1 duplication iPSCs lines, showing normal karyotype (46), without detectable chromosomal.** Scale bar: 10 µm.

**
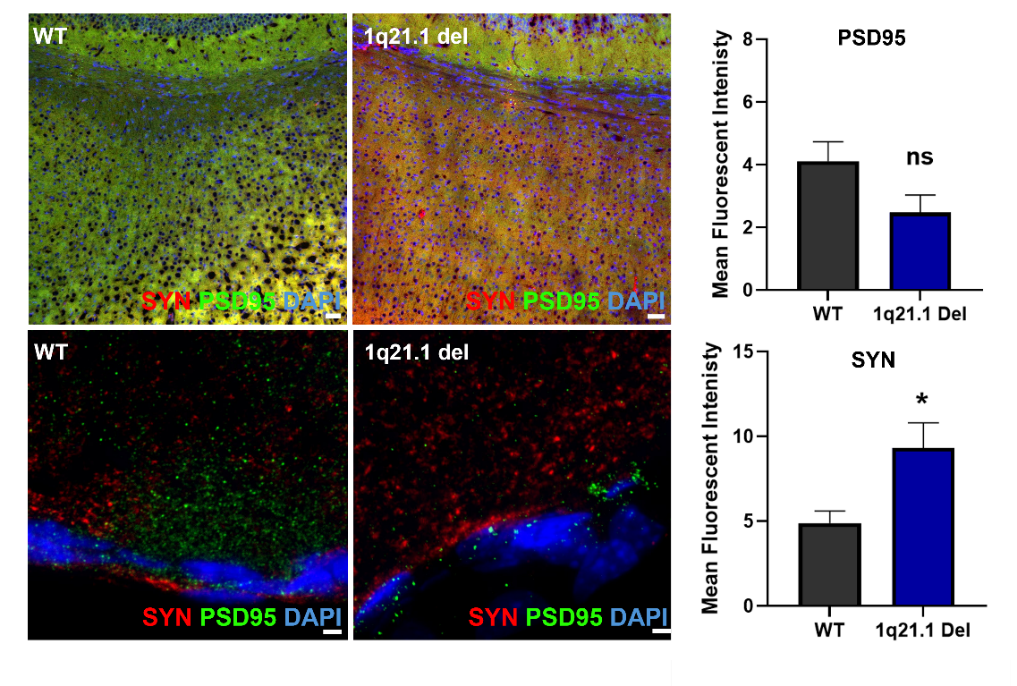
**

**Supplementary Figure 4. Reduced Synaptophysin (SYN) and Post-synaptic density 95 (PSD95) marker expression in frontal cortex of mouse carrying 1q21.1 deletion.** The fluorescent images visualize SYN (red) and PSD95 (green) in 4-weeks old mouse brain, counterstained with DAPI (blue). Top images scale bar: 50 µm, bottom images scale bar = 5 µm. The bar graph illustrates PSD95, and SYN mean fluorescence intensity analysis of the immunofluorescent signal. Data are represented as Control N=5 and 1q21.1 deletion N=5. Statistical analysis was performed using unpaired t-test comparing the deletion to the control. Significance levels were denoted as ns p > 0.05, * p< 0.05, ** p < 0.002, *** p < 0.0002, and **** p < 0.0001.


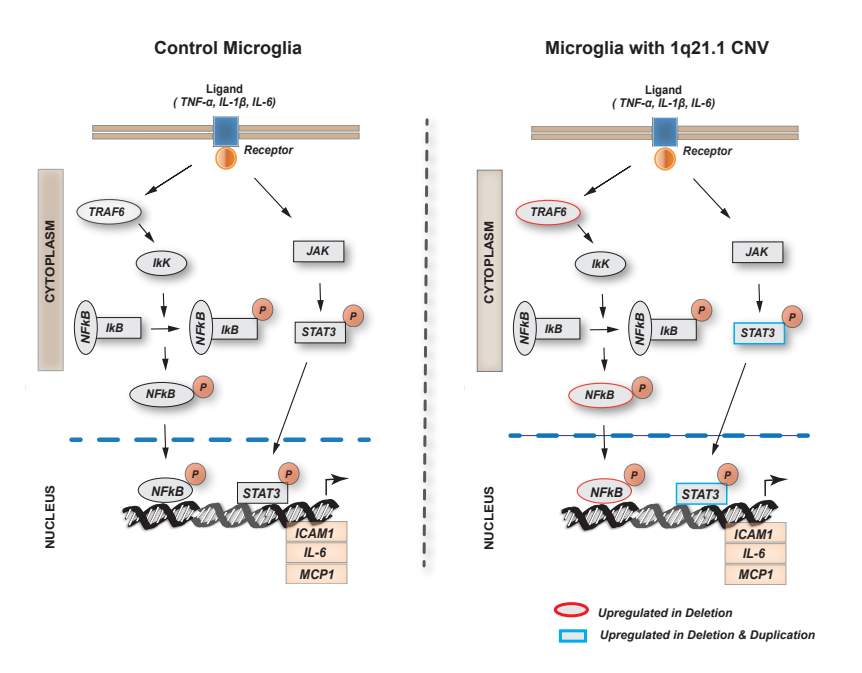


**Supplementary Figure 5. The NF-κB and STAT3 signaling pathways may potentially collaborate to connect immune dysfunction in microglia with 1q21.1 deletion and duplication.** Through immune gene pathway analysis, we hypothesize that NF-κB signaling is activated in microglia with 1q21.1 deletion by Toll-like receptors (TLRs) and TLR ligands, as well as cytokines like IL-6, IL-1B, and TNF-α. This activation leads to the ubiquitination of TRAF6, which in turn phosphorylates NF-κB. On the other hand, the activation of JAK/STAT signalling occurs in microglia carrying a duplication of the 1q21.1 locus following binding cytokines such as IL-6, IL-1B, and TNF-α to the receptor. The phosphorylation of both NFκB and STAT3 results in differential expression of immune genes, including NLRP3, Pro-IL-1β, Pro-IL-18, IL-6, MCP1, and ICAM1 genes, in microglia with 1q21.1 deletion and duplication in comparison to control microglia.

**Supplementary
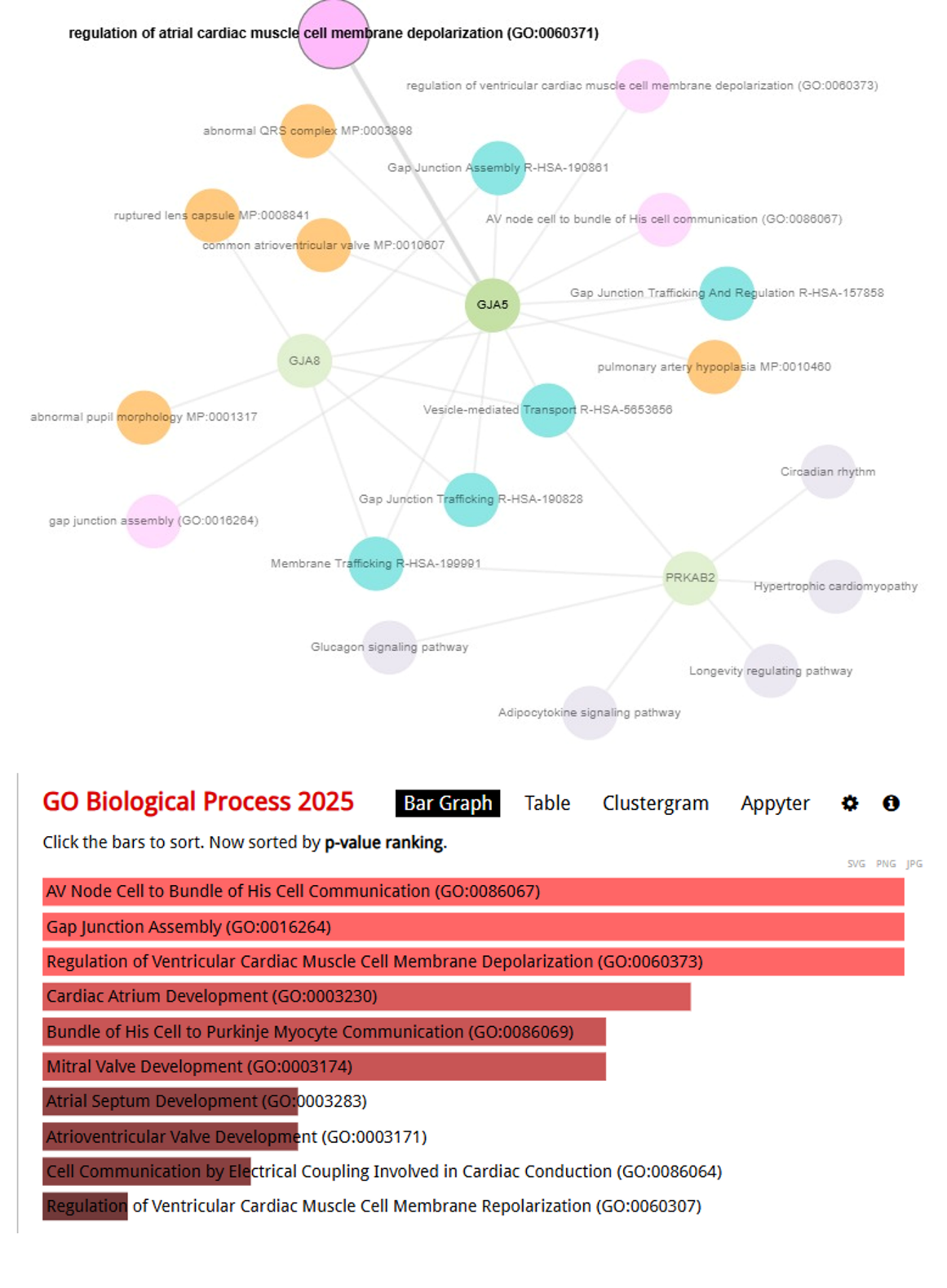
Figure 6. Gene ontology enrichment analysis revealing no single gene association towards inflammatory pathways.** Network representation (top) highlights GJA5 genes as central hub connected to key pathways. Bar graph (bottom) visualizes top-ranked GO biological processes sorted by enrichment significance.

**Material and Methods Supplementary Tables**

**Table S1. Patient information for all 1q21.1 patients used in the study**. GAD: generalized anxiety disorder; MDD: major depressive; OCD: obsessive compulsive disorder; PD: personality disorder.

| ***1q21.1 CNV*** | ***Sex*** | ***Full IQ*** | ***Clinical Symptom*** | ***Medication*** | ***1q21.1 Mutation Coordinates*** | ***Other CNVs*** |
| --- | --- | --- | --- | --- | --- | --- |
| ***Deletion 1*** | Male | 77 | Agoraphobia, avoidant PD, obsessive-compulsive PD, depressive PD, other substance related disorder, conduct disorder | N/A | chr1:146496661-  147375981 | chr12:2027625-  2550818_dup |
| ***Deletion 2*** | Female | 78 | Adjustment Disorder, cardiac: deformed valve, narrowing of aorta, thyroid problems, hearing difficulties, sleep apnoea | Citalopram | chr1:146330584- 147825662 | chr1:63918079- 64038174_dup, chr5:19503096- 19985234_del |
| ***Deletion 3*** | Male | 109 | Social phobia, OCD, MDD, avoidant PD; interstitial cystitis (daily catheterisation), short stature, recurrent infections; tremor | Propranolol | chr1:146496661- 147391614 | N/A |
| ***Duplication 1*** | Female | 89 | Social phobia, GAD, MDD, avoidant PD, conduct disorder, hearing difficulties | N/A | chr1:146330584- 146982763 | N/A |
| ***Duplication 2*** | Female | 109 | Social phobia, panic disorder, agoraphobia, OCD, MDD, psychotic symptoms, schizotypal PD, Borderline PD; osteoarthritis, visual difficulties; | Propranolol | chr1:146330584- 147825662 | N/A |
| ***Duplication 3*** | Male | 116 | Autism Spectrum Disorder |  | chr1:146326373-147385641 | N/A |

**Table S2. Microglial differentiation media (MGdM) components**

| ***Microglia Differentiation Media (MGdM)*** | |
| --- | --- |
| Neurobasal (Gibco™) | Basal Media |
| 1X Gem21 (without vitamin A, Gemini Bio) | Supplements |
| 1X N2 supplement (Gibco™) |  |
| 85% syrup stock, Lactic acid (Sigma-Aldrich) |  |
| 1 mM Sodium Pyruvate (Sigma-Aldrich) |  |
| 1X GlutaMAX™ (Thermofisher) |  |
| 3.5 ng/ml Biotin (Sigma-Aldrich) |  |
| 2 mg/ml Lipidated BSA (Albumax I, Gibco™) |  |
| 2.5 ug/ml Ascorbic Acid (Sigma-Aldrich) |  |
| 1X Antibiotics and antimycotics (Sigma-Aldrich) |  |
| 5M NaCl (Sigma-Aldrich) |  |

**Table S3. List of antibodies used in the study**.

| ***Antibody*** | ***Dilution*** | ***Manufacturer*** |  | ***Catalogue number*** |
| --- | --- | --- | --- | --- |
| CD-40 (Host: Mouse) | 1:250 | eBioscience |  | 14-0409-82 |
| IBA1 (Host: Rabbit) | 1:250 | Wako |  | 019-19741 |
| TNF-α (Host: Rabbit) | 1:250 | Novus |  | NBP119532 |
| PU.1 (Host: Rabbit) | 1:250 | Abcam |  | ab183327 |
| SYN (Host: Mouse) | 1:400 | Abcam |  | ab8049 |
| PSD95 (Host: Rabbit) | 1:800 | Abcam |  | ab18258 |
| 555-Donkey Anti-Mouse | 1:2000 | Thermofisher |  | A31570 |
| 555-Donkey Anti-Rabbit | 1:2000 | Thermofisher |  | A31572 |

**Table S4. List of qRT-PCR primers, alignments of Forward and Reverse primer sequences are 5’ to 3’.**

| ***Gene*** | ***Forward Primer*** | ***Reverse Primer*** | ***Source*** |
| --- | --- | --- | --- |
| GAPDH | CTGGTAAAGTGGATATTGTTGCCAT | TGGAATCATATTGGAACATGTAAACC | PrimerBLAST |
| IL-1B | CCACAGACCTTCCAGGAGAATG | GTGCAGTTCAGTGATCGTACAGG | PrimerBLAST |
| MCP-1 | AGAATCACCAGCAGCAAGTGTCC | TCCTGAACCCACTTCTGCTTGG | PrimerBLAST |
| MMP-2 | AGCGAGTGGATGCCGCCTTTAA | CATTCCAGGCATCTGCGATGAG | PrimerBLAST |

**Table S5. Gene table for RT² Profiler PCR Array: PAHS-052Z (RT² Profiler™ PCR Array Human Innate & Adaptive Immune Responses)**

|  |  | |  |  |  | |
| --- | --- | --- | --- | --- | --- | --- |
| **Ref seq** | | **Description** | | | | **Gene name** |
| NM_001639 | | Amyloid P component, serum | | | | HEL-S-92n/PTX2/SAP |
| NM_000064 | | Complement component 3 | | | | AHUS5/ARMD9/ASP/C3a/C3b/CPAMD1/HEL-S-62p |
| NM_033292 | | Caspase 1, apoptosis-related cysteine peptidase (interleukin 1, beta, convertase) | | | | ICE/IL1BC/P45 |
| NM_002982 | | Chemokine (C-C motif) ligand 2 | | | | GDCF-2/HC11/HSMCR30/MCAF/MCP-1/MCP1/SCYA2/SMC-CF |
| NM_002985 | | Chemokine (C-C motif) ligand 5 | | | | D17S136E/RANTES/SCYA5/SIS-delta/SISd/TCP228/eoCP |
| NM_005508 | | Chemokine (C-C motif) receptor 4 | | | | CC-CKR-4/CD194/CKR4/CMKBR4/ChemR13/HGCN:14099/K5-5 |
| NM_000579 | | Chemokine (C-C motif) receptor 5 | | | | CC-CKR-5/CCCKR5/CCR-5/CD195/CKR-5/CKR5/CMKBR5/IDDM22 |
| NM_004367 | | Chemokine (C-C motif) receptor 6 | | | | BN-1/C-C CKR-6/CC-CKR-6/CCR-6/CD196/CKR-L3/CKRL3/CMKBR6/DCR2/DRY6/GPR29/GPRCY4/STRL22 |
| NM_005201 | | Chemokine (C-C motif) receptor 8 | | | | CC-CKR-8/CCR-8/CDw198/CKRL1/CMKBR8/CMKBRL2/CY6/GPRCY6/TER1 |
| NM_000591 | | CD14 molecule | | | | - |
| NM_000616 | | CD4 molecule | | | | CD4mut |
| NM_001250 | | CD40 molecule, TNF receptor superfamily member 5 | | | | Bp50/CDW40/TNFRSF5/p50 |
| NM_000074 | | CD40 ligand | | | | CD154/CD40L/HIGM1/IGM/IMD3/T-BAM/TNFSF5/TRAP/gp39/hCD40L |
| NM_005191 | | CD80 molecule | | | | B7/B7-1/B7.1/BB1/CD28LG/CD28LG1/LAB7 |
| NM_006889 | | CD86 molecule | | | | B7-2/B7.2/B70/CD28LG2/LAB72 |
| NM_001768 | | CD8a molecule | | | | CD8/Leu2/MAL/p32 |
| NM_000567 | | C-reactive protein, pentraxin-related | | | | PTX1 |
| NM_000758 | | Colony stimulating factor 2 (granulocyte-macrophage) | | | | GMCSF |
| NM_001565 | | Chemokine (C-X-C motif) ligand 10 | | | | C7/IFI10/INP10/IP-10/SCYB10/crg-2/gIP-10/mob-1 |
| NM_001504 | | Chemokine (C-X-C motif) receptor 3 | | | | CD182/CD183/CKR-L2/CMKAR3/GPR9/IP10-R/Mig-R/MigR |
| NM_014314 | | DEAD (Asp-Glu-Ala-Asp) box polypeptide 58 | | | | RIG-I/RIGI/RLR-1/SGMRT2 |
| NM_000639 | | Fas ligand (TNF superfamily, member 6) | | | | ALPS1B/APT1LG1/APTL/CD178/CD95-L/CD95L/FASL/TNFSF6 |
| NM_014009 | | Forkhead box P3 | | | | AIID/DIETER/IPEX/JM2/PIDX/XPID |
| NM_002051 | | GATA binding protein 3 | | | | HDR/HDRS |
| NM_002116 | | Major histocompatibility complex, class I, A | | | | HLAA |
| NM_005516 | | Major histocompatibility complex, class I, E | | | | EA1.2/EA2.1/HLA-6.2/MHC/QA1 |
| NM_000201 | | Intercellular adhesion molecule 1 | | | | BB2/CD54/P3.58 |
| NM_024013 | | Interferon, alpha 1 | | | | IFL/IFN/IFN-ALPHA/IFN-alphaD/IFNA13/IFNA@ |
| NM_000629 | | Interferon (alpha, beta and omega) receptor 1 | | | | AVP/IFN-alpha-REC/IFNAR/IFNBR/IFRC |
| NM_002176 | | Interferon, beta 1, fibroblast | | | | IFB/IFF/IFN-beta/IFNB |
| NM_000619 | | Interferon, gamma | | | | IFG/IFI |
| NM_000416 | | Interferon gamma receptor 1 | | | | CD119/IFNGR/IMD27A/IMD27B |
| NM_000572 | | Interleukin 10 | | | | CSIF/GVHDS/IL-10/IL10A/TGIF |
| NM_002188 | | Interleukin 13 | | | | IL-13/P600 |
| NM_002190 | | Interleukin 17A | | | | CTLA-8/CTLA8/IL-17/IL-17A/IL17 |
| NM_001562 | | Interleukin 18 (interferon-gamma-inducing factor) | | | | IGIF/IL-18/IL-1g/IL1F4 |
| NM_000575 | | Interleukin 1, alpha | | | | IL-1A/IL1/IL1-ALPHA/IL1F1 |
| NM_000576 | | Interleukin 1, beta | | | | IL-1/IL1-BETA/IL1F2 |
| NM_000877 | | Interleukin 1 receptor, type I | | | | CD121A/D2S1473/IL-1R-alpha/IL1R/IL1RA/P80 |
| NM_000586 | | Interleukin 2 | | | | IL-2/TCGF/lymphokine |
| NM_016584 | | Interleukin 23, alpha subunit p19 | | | | IL-23/IL-23A/IL23P19/P19/SGRF |
| NM_000589 | | Interleukin 4 | | | | BCGF-1/BCGF1/BSF-1/BSF1/IL-4 |
| NM_000879 | | Interleukin 5 (colony-stimulating factor, eosinophil) | | | | EDF/IL-5/TRF |
| NM_000600 | | Interleukin 6 (interferon, beta 2) | | | | BSF2/HGF/HSF/IFNB2/IL-6 |
| NM_000584 | | Interleukin 8 | | | | GCP-1/GCP1/IL8/LECT/LUCT/LYNAP/MDNCF/MONAP/NAF/NAP-1/NAP1 |
| NM_001569 | | Interleukin-1 receptor-associated kinase 1 | | | | IRAK/pelle |
| NM_001571 | | Interferon regulatory factor 3 | | | | - |
| NM_001572 | | Interferon regulatory factor 7 | | | | IMD39/IRF-7H/IRF7A/IRF7B/IRF7C/IRF7H |
| NM_000632 | | Integrin, alpha M (complement component 3 receptor 3 subunit) | | | | CD11B/CR3A/MAC-1/MAC1A/MO1A/SLEB6 |
| NM_004972 | | Janus kinase 2 | | | | JTK10/THCYT3 |
| NM_015364 | | Lymphocyte antigen 96 | | | | ESOP-1/MD-2/MD2/ly-96 |
| NM_000239 | | Lysozyme | | | | LZM |
| NM_002745 | | Mitogen-activated protein kinase 1 | | | | ERK/ERK-2/ERK2/ERT1/MAPK2/P42MAPK/PRKM1/PRKM2/p38/p40/p41/p41mapk/p42-MAPK |
| NM_002750 | | Mitogen-activated protein kinase 8 | | | | JNK/JNK-46/JNK1/JNK1A2/JNK21B1/2/PRKM8/SAPK1/SAPK1c |
| NM_000242 | | Mannose-binding lectin (protein C) 2, soluble | | | | COLEC1/HSMBPC/MBL/MBL2D/MBP/MBP-C/MBP1/MBPD |
| NM_000250 | | Myeloperoxidase | | | | - |
| NM_002462 | | Myxovirus (influenza virus) resistance 1, interferon-inducible protein p78 (mouse) | | | | IFI-78K/IFI78/MX/MxA |
| NM_002468 | | Myeloid differentiation primary response gene (88) | | | | MYD88D |
| NM_003998 | | Nuclear factor of kappa light polypeptide gene enhancer in B-cells 1 | | | | EBP-1/KBF1/NF-kB1/NF-kappa-B/NF-kappaB/NFKB-p105/NFKB-p50/NFkappaB/p105/p50 |
| NM_020529 | | Nuclear factor of kappa light polypeptide gene enhancer in B-cells inhibitor, alpha | | | | IKBA/MAD-3/NFKBI |
| NM_183395 | | NLR family, pyrin domain containing 3 | | | | AGTAVPRL/AII/AVP/C1orf7/CIAS1/CLR1.1/FCAS/FCAS1/FCU/MWS/NALP3/PYPAF1 |
| NM_006092 | | Nucleotide-binding oligomerization domain containing 1 | | | | CARD4/CLR7.1/NLRC1 |
| NM_022162 | | Nucleotide-binding oligomerization domain containing 2 | | | | ACUG/BLAU/CARD15/CD/CLR16.3/IBD1/NLRC2/NOD2B/PSORAS1 |
| NM_000448 | | Recombination activating gene 1 | | | | RAG-1/RNF74 |
| NM_005060 | | RAR-related orphan receptor C | | | | NR1F3/RORG/RZR-GAMMA/RZRG/TOR |
| NM_000578 | | Solute carrier family 11 (proton-coupled divalent metal ion transporters), member 1 | | | | LSH/NRAMP/NRAMP1 |
| NM_007315 | | Signal transducer and activator of transcription 1, 91kDa | | | | CANDF7/IMD31A/IMD31B/IMD31C/ISGF-3/STAT91 |
| NM_003150 | | Signal transducer and activator of transcription 3 (acute-phase response factor) | | | | ADMIO/APRF/HIES |
| NM_003151 | | Signal transducer and activator of transcription 4 | | | | SLEB11 |
| NM_003153 | | Signal transducer and activator of transcription 6, interleukin-4 induced | | | | D12S1644/IL-4-STAT/STAT6B/STAT6C |
| NM_013351 | | T-box 21 | | | | T-PET/T-bet/TBET/TBLYM |
| NM_182919 | | Toll-like receptor adaptor molecule 1 | | | | IIAE6/MyD88-3/PRVTIRB/TICAM-1/TRIF |
| NM_003263 | | Toll-like receptor 1 | | | | CD281/TIL/TIL. LPRS5/rsc786 |
| NM_003264 | | Toll-like receptor 2 | | | | CD282/TIL4 |
| NM_003265 | | Toll-like receptor 3 | | | | CD283/IIAE2 |
| NM_138554 | | Toll-like receptor 4 | | | | ARMD10/CD284/TLR-4/TOLL |
| NM_003268 | | Toll-like receptor 5 | | | | MELIOS/SLE1/SLEB1/TIL3 |
| NM_006068 | | Toll-like receptor 6 | | | | CD286 |
| NM_016562 | | Toll-like receptor 7 | | | | TLR7-like |
| NM_138636 | | Toll-like receptor 8 | | | | CD288 |
| NM_017442 | | Toll-like receptor 9 | | | | CD289 |
| NM_000594 | | Tumor necrosis factor | | | | DIF/TNF-alpha/TNFA/TNFSF2 |
| NM_004620 | | TNF receptor-associated factor 6 | | | | MGC:3310/RNF85 |
| NM_003331 | | Tyrosine kinase 2 | | | | IMD35/JTK1 |
| NM_001101 | | Actin, beta | | | | BRWS1/PS1TP5BP1 |
| NM_004048 | | Beta-2-microglobulin | | | | - |
| NM_002046 | | Glyceraldehyde-3-phosphate dehydrogenase | | | | G3PD/GAPD/HEL-S-162eP |
| NM_000194 | | Hypoxanthine phosphoribosyltransferase 1 | | | | HGPRT/HPRT |
| NM_001002 | | Ribosomal protein, large, P0 | | | | L10E/LP0/P0/PRLP0/RPP0 |
| SA_00105 | | Human Genomic DNA Contamination | | | | HIGX1A |
| SA_00104 | | Reverse Transcription Control | | | | RTC |
| SA_00104 | | Reverse Transcription Control | | | | RTC |
| SA_00104 | | Reverse Transcription Control | | | | RTC |
| SA_00103 | | Positive PCR Control | | | | PPC |
| SA_00103 | | Positive PCR Control | | | | PPC |
| SA_00103 | | Positive PCR Control | | | | PPC |
